# Radiation dose considerations in digital radiography with an anti‐scatter grid: A study using adult and pediatric phantoms

**DOI:** 10.1002/acm2.14081

**Published:** 2023-07-25

**Authors:** Hiroki Kawashima, Katsuhiro Ichikawa, Azusa Kitao, Takashi Matsubara, Takumi Sugiura, Tomohiro Kobayashi, Satoshi Kobayashi

**Affiliations:** ^1^ Faculty of Health Sciences, Institute of Medical, Pharmaceutical and Health Sciences Kanazawa University Kanazawa Japan; ^2^ Department of Radiology Kanazawa University Graduate School of Medical Sciences Kanazawa Japan

**Keywords:** anti‐scatter grid, digital radiography, phantom study, radiation dose

## Abstract

**Background:**

When using an anti‐scatter grid, a decrease in receptor dose caused by its X‐ray absorption seems to lead to the misperception that radiation dose needs to be increased even in digital radiography (DR).

**Objective:**

To demonstrate that there is no need to increase radiation dose in DR with a grid, based on a visual evaluation using an adult and a pediatric abdomen phantom (P_AD_ and P_PD_, respectively).

**Materials and methods:**

Phantom images with and without a grid were obtained with exposure parameters determined based on a preliminarily measured signal‐to‐noise ratio improvement factor (SIF), an index for potential dose reduction when using a grid. In visual evaluation, four radiologists compared phantom images with a grid applied at different dose reduction rates (0% [no reduction], 18%, 36%, and 59% for P_AD_ and 0% and 11% for P_PD_) against an image without a grid at the baseline dose (as the reference). They graded the overall image quality of the former relative to that of the latter (reference) on a 3‐point scale (3 = better, 2 = almost equal, 1 = worse).

**Results:**

The mean scores for dose reduction rates of 0%, 18%, 36%, and 59% were 3.00, 3.00, 2.75, and 1.00, respectively, for P_AD_; those for 0% and 11% were 2.13 and 1.63, respectively, for P_PD_. These results support the validity of our view that no dose increase is necessary when using an anti‐scatter grid. Actually, there is even a potential for improvement in image quality with dose reduction rates of ≤36% for P_AD_.

**Conclusion:**

It is worth reconsidering the necessity of increasing radiation dose in the DR imaging of the adult and pediatric abdomens with an anti‐scatter grid.

## 1 INTRODUCTION

The use of an anti‐scatter grid is one of the most widely practiced methods to control the amount of scattered radiation in diagnostic X‐ray imaging.^1^ The grid is physically placed between the patient and the receptor and works to reduce scattered radiation before it reaches the receptor; thus, the use of one helps to recover the radiographic contrast degraded by scatter radiation. However, since the grid reduces not only scattered radiation but also some primary radiation, it decreases the total amount of radiation reaching the receptor compared with grid‐less acquisition.

This decrease in receptor dose has unfortunately led to the wide‐spread misperception that radiation dose needs to be increased also in digital radiography (DR) when an anti‐scatter grid is applied, as was the case with analog radiography. The notion that radiation dose to the patient needs to be increased when a grid is used^2^, ^3^, ^4^, ^5^, ^6^ seems to focus on maintaining receptor exposure between the two cases: with and without the grid. For example, based on this notion, while the imaging (with or without a grid) was performed depending on the situation in portable radiography for adult abdomens, the exposure parameters might be adjusted according to the bucky factor (BF), without considering the differences in image quality optimization method between analog and digital systems.

In screen‐film (analog) systems, since the optical density decreases with the dose decrease caused by the grid, a dose increase is mandatory to ensure adequate optical density. On the other hand, in DR, image brightness does not depend on radiation dose; thus, there is no need to maintain the receptor exposure when using a grid.^7^, ^8^, ^9^ This fundamental property of the grid in DR has been proven in previous studies. Furthermore, the amount of dose can (could) be reduced compared with grid‐less acquisition in most cases.^10^, ^11^, ^12^, ^13^, ^14^ They mostly use the signal‐to‐noise ratio improvement factor (SIF) to demonstrate how much the signal‐to‐noise ratio (SNR) is improved by a grid.^10^, ^11^, ^12^ These are the phantom studies simulating different subject thicknesses achieved by polymethyl methacrylate (PMMA) slabs. However, to the best of our knowledge, the benefit of using a grid has not been sufficiently demonstrated with cases simulating clinical situations more suitably than using PMMA slabs.

The purpose of this study is to demonstrate that dose increase is unnecessary in DR with an anti‐scatter grid, by presenting the results of a visual evaluation of images of two anthropomorphic phantoms that simulate an adult and a pediatric body.

## 2 METHODS

### 2.1 Anthropomorphic phantom

**FIGURE 1:**
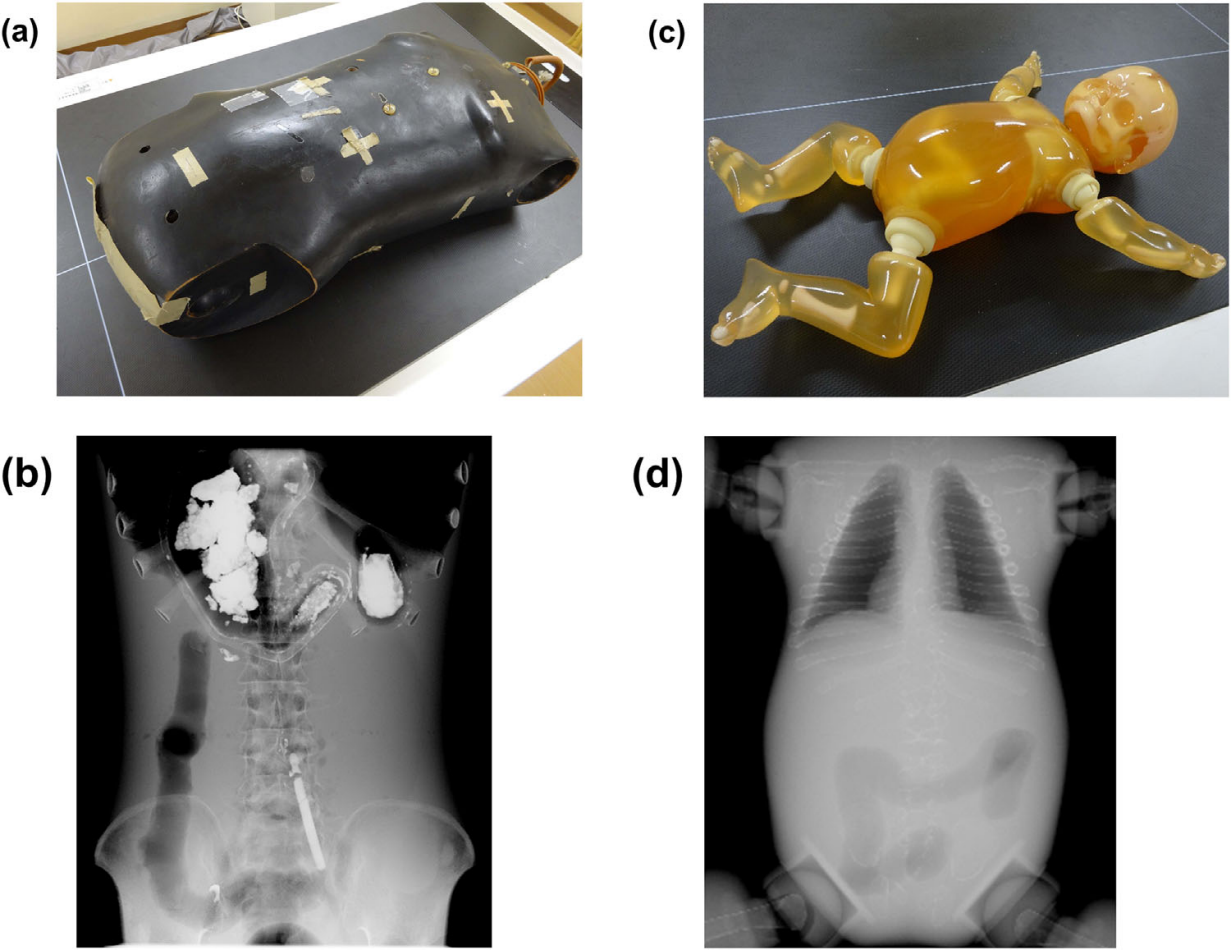
Photographs and sample radiographs of the adult (a, b) and pediatric (c, d) phantoms.

Two anthropomorphic phantoms were used in this study (Figure 1): one simulating an adult body torso (chest to pelvis), and the other simulating a whole pediatric body. The former contains dry bones (ribs, spines, and pelvic bones) and air cavities simulating lungs, the stomach, and the colon; the latter contains artificial bones and air cavities simulating lungs and the colon. The maximum thicknesses of abdominal parts are 200 mm for the adult body torso phantom (adult phantom) and 100 mm for the pediatric whole‐body phantom (pediatric phantom). In this study with the adult phantom, a portable abdominal radiography was assumed because there could be both cases with and without a grid.

### 2.2 Grid and imaging system

Two aluminum interspace anti‐scatter grids with grid ratios of 10:1 (G10) and 6:1 (G6) (Mitaya Manufacturing, Saitama, Japan) were used. Both grids have 40 lines per centimeter, and a focal distance of 1200 mm. All the images were obtained by an indirect‐type flat panel detector (FPD) Calneo Smart (Fujifilm, Tokyo, Japan) with a cesium iodide scintillator. The FPD has a pixel size of 0.15 mm and a matrix size of 2336 × 2836 pixels. Phantom exposures were performed with an inverter‐type X‐ray unit RADspeed Pro (Shimadzu, Kyoto, Japan). Its X‐ray tube system has a total equivalent filtration of 2.8 mm aluminum. The raw image data, which were saved using a raw‐data‐saving function implemented in the operation software of the FPD system, was used for data analysis. With this function, the manufacturer guarantees that the data have a precise linearity and is not processed by any filtering. In cases with the grid, only grid‐line removal processing, which filters out specific frequency components of the grid‐line artifacts,^15^ was additionally applied before saving the raw data, without affecting the linearity.

### 2.3 Pre‐investigation to measure SIF

To determine the dose ranges for imaging the adult and pediatric phantoms, the following pre‐investigation was performed to measure the SIFs for G10 and G6.

### 2.4 Measurement of SIF

**FIGURE 2:**
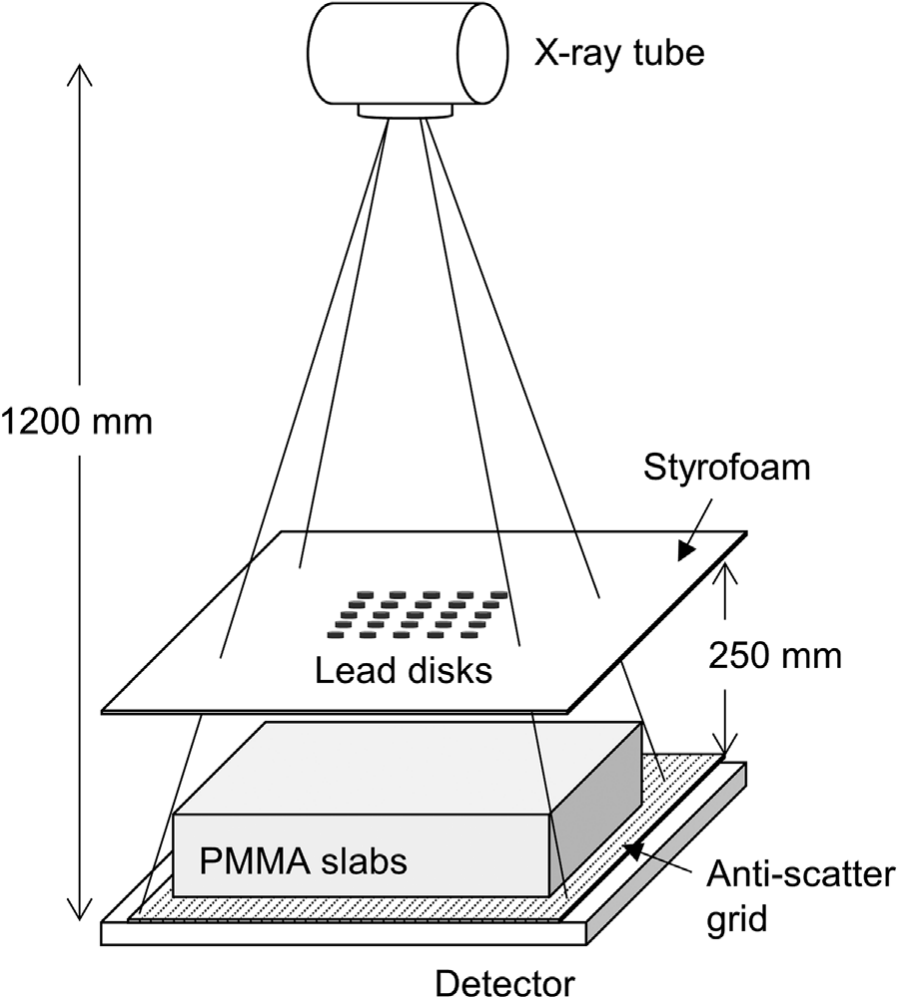
Geometric arrangement for measuring the scatter fraction (SF) and the bucky factor (BF).

Figure 2 shows the geometric arrangement for the SIF measurement. SIFs were measured for the thicknesses of 200 and 100 mm corresponding to the maximum thicknesses of the adult and pediatric phantoms, using PMMA slabs having a cross‐sectional area of 350 × 350 mm^2^ and a thickness of 50‐mm. Since the materials are different between the anthropomorphic phantoms and the PMMA slabs, the SIFs do not precisely match between the materials with the same thickness. However, PMMA is now widely used to simulate the soft tissue for measuring the SIF; therefore, approximated SIFs were estimated using PMMA in this study. The details of the beam stop array are as follows: an array of 5 × 5 cylinders, each of which is 3 mm in diameter and 6 mm in height, embedded in a 6‐mm sheet of styrofoam, and is spaced 25‐mm apart from each other. This array was positioned at 250 mm above the detector surface, which ensured a uniform magnification of the beam stop. The grids of G10 and G6 were used for the thicknesses of 200 and 100 mm, respectively.

According to the exposure condition clinically used in our hospital, combinations of the tube voltage and the field of view (FOV) were set at 80 kVp and 350 × 350 mm^2^ at the detector surface (large FOV) and at 60 kVp and 200 × 200 mm^2^ (small FOV) for PMMA thicknesses of 200 and 100 mm. Scatter fractions (*SFs*) with and without a grid and *BFs* were measured by previously reported procedures.^10^, ^16^, ^17^ Averaged pixel values were measured from a region of interest (ROI) 10 pixels in diameter placed at each beam stop and on the back‐ground next to the beam stop. The *SF* was determined by calculating the ratio of these ROI values using the formula below.

(1) $$SF\;=\;\frac{ROI_{beam\;stop}}{ROI_{background}}$$

The BF can be also calculated from background ROI values with and without the grid.

By using the *SF* and *BF* thus obtained, the SIF was calculated as follows:

(2) $$SIF\;=\;\frac{1-SF_{G}}{1-SF_{nonG}}\cdot \frac{1}{\sqrt{BF}}$$

where *SF_G_* and *SF_nonG_* denote scatter fractions with and without a grid, respectively. By using the scatter‐to‐primary ratio (SPR), “(*1 + SPR_nonG_*)/(*1 + SPR_G_*)” can be used instead of the “(*1−SF_G_*)/(*1‐SF_nonG_*)” in Equation (2). SIF > 1.0 means that the SNR is improved by using a grid compared with grid‐less acquisition at the same incident dose.

### 2.5 Resultant SIF

The SIF value was 1.54 (*SF_G_* = 43.0%, *SF_nonG_* = 84.7%, *BF* = 5.87) for the thickness of 200 mm at 80 kVp with the large FOV corresponding to the adult phantom. For the thickness of 100 mm at 60 kVp with the small FOV corresponding to the pediatric phantom, the SIF value was 1.09 (*SF_G_* = 22.4%, *SF_nonG_* = 59.2%, *BF* = 3.04). Since the SIF can be interpreted as the ratio of the SNR with a grid to the SNR without a grid, these results imply that the use of a grid can improve the SNR, or conversely, that the radiation dose can be reduced with the same SNR. According to the principle that SNR^2^ is proportional to radiation dose,^14^ the dose reduction rate, which is be defined as (1 − 1/*SIF* ^2^) × 100%,^10^ was 58% and 16% for the thicknesses of 200 and 100 mm, respectively.

### 2.6 Exposure parameters for anthropomorphic phantom

The tube voltage was set at 80 kVp with G10 and 60 kVp with G6 for the adult and pediatric phantoms, respectively, as in the pre‐investigation. The baseline milliampere second (mAs) value was set at 22 and 3.6 for the adult and pediatric phantoms, respectively, considering the diagnostic reference level^18^ and the exposure condition in our hospital. The resultant entrance surface air kerma for the adult and pediatric phantoms was 1.4 and 0.10 mGy, respectively, which were measured using a 6‐cc ionization chamber (Model 10 × 6‐6, Radcal, Monrovia, CA, USA) and an electrometer (Accu‐Gold, Radcal) placed in free‐in‐air conditions.

The reduced mAs values with a grid applied were chosen to be 9, 14, and 18 mAs (i.e., 59%, 36%, and 18% dose reductions) for the adult phantom. First, the maximum dose reduction rate was determined based on the SIF value, and then below this maximum, these mAs values were chosen to investigate how the reduction in radiation dose affects the results. The mAs value chosen to be 3.2 (i.e., 11% dose reduction) for the pediatric phantom. The exposure was repeated two times for each combination of exposure parameters to increase the number of images for visual evaluation described in the next subsection.

### 2.7 Visual evaluation

A relative visual grading was performed to assess the image quality when using a grid, in comparison with that when not using a grid, while observing the anthropomorphic phantoms. The image without a grid of the baseline mAs value was kept on the left side on a medical‐grade display as the reference image. Other images with a grid applied were displayed randomly on the right side one by one while hiding the dose reduction rate. A total of eight image pairs (four doses × 2) and a total of four image pairs (two doses × 2) were used for the adult and pediatric phantoms, respectively. The images were trimmed to 600 × 700 pixels at a portion on the left side of the upper abdomen for the adult phantom and an abdominal portion centered on the lumber spine for the pediatric phantom. The trimming was performed because it was hard to equalize the display contrasts of the entire abdomen between the two cases (with and without a grid) due to a notable difference in scatter fraction. The left side of the upper abdomen for the adult phantom was suitable for the evaluation because it contained a small gas shadow and had a relatively adjustable display contrast. Trimming was also convenient to eliminate the effect of any image interpolation for displaying. The window condition was adjusted so that the contrast between the lumber vertebra and the soft tissues surrounding it was the same in the image pair. The order of displaying image pairs of each phantom was randomized. Four radiologists (A.K., T.M., T.S., and T.K. with 18, 13, 9, and 7 years of clinical experience, respectively) graded overall quality of images with a grid relative to that without a grid using a 3‐point scale, where 3 = better, 2 = almost equal, and 1 = worse. Overall image quality was judged by mainly the differences in the image noise because the display contrast was already adjusted. The radiologists were not allowed to change the window conditions. This restriction would be reasonable for the visual evaluation based on the optimization of DR.^7^ The grading time was not limited.

The Fleiss' kappa test was used to assess the degree of agreement between the radiologists. The kappa value was interpreted as follows: ≤0.20 = poor, 0.21–0.40 = fair, 0.41–0.6 = moderate, 0.61–0.8 = good, and 0.81–1.00 = excellent agreement. The image quality scores were compared by using the Wilcoxon signed‐rank test with Bonferroni correction (*n* = 4 for the adult abdomen) or without correction (for the pediatric abdomen). The *P* values of 0.0125 and 0.05 or less were regarded as indicating a statistically significant difference for the adult and pediatric abdomens, respectively. A statistical analysis was performed using the commercially available software SPSS for Windows version 25 (IBM, Armonk, NY, USA).

## 3 RESULTS

### 3.1 Image quality score

**FIGURE 3:**
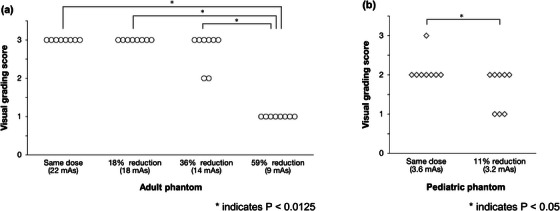
Plots of image quality scores at each dose level for (a) the adult and (b) the pediatric phantoms. Four radiologists graded the overall quality of images with a grid applied relative to that without a grid, using a 3‐point scale.

Figure 3 shows the results of the visual evaluation (visual score) that compared the images with a grid applied against those without a grid for the adult and pediatric phantoms. There was good concordance between the four radiologists because the kappa coefficient was 0.78. For the adult phantom, all the scores of the images at the same incident dose were 3.00, which means that the quality of the visual images with a grid applied was superior to that without a grid. The mean scores for 18%, 36%, and 59% dose reductions were 3.00, 2.75, and 1.00, respectively. The score for the 59% dose reduction was significantly lower than the others. There was no score of 1 (=inferior) for the dose reduction rate ≤36%. For the pediatric phantom, the mean score at the same incident dose was 2.13, which means that the quality of the visual images with a grid applied was almost equal to that without a grid. In contrast, the mean score for the 11% dose reduction was 1.63, significantly lower than that at the same incident dose. The scores for the 11% dose reduction included three instances of one where the total was eight.

### 3.2 Phantom image impressions

**FIGURE 4:**
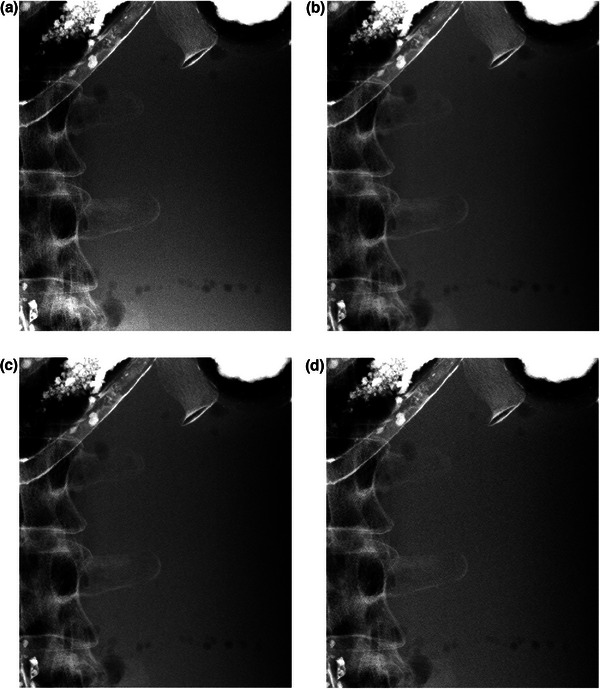
Images of the adult phantom used in visual grading analysis: (a) without a grid, (b) with a grid applied at the same incident dose (0% dose reduction), (c) with a grid applied with 36% dose reduction, and (d) with a grid applied with 59% dose reduction.

Figure 4 shows images of the adult phantom without a grid and those with a grid applied at 0% (the same incident dose), 36%, and 58% dose reductions. The visual evaluators summarize their subjective impressions as follows. Although the appearance of the lumbar vertebra was almost the same, the image noise with a grid applied was less at the same incident dose (Figure 4b), slightly less (or comparable) at the 36% dose reduction (Figure 4c), and quite notable at the 58% dose reduction (Figure 4d), compared with the case without a grid.

**FIGURE 5:**
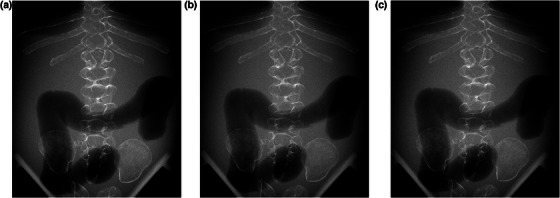
Images of the pediatric phantom used in visual grading analysis: (a) without a grid, (b) with a grid applied at the same incident dose (0% dose reduction), and (c) with a grid applied with 11% dose reduction.

Figure 5 shows images of the pediatric phantom without a grid and those with a grid applied at 0% (the same incident dose) and 11% dose reduction. Although the difference in visual image quality was not very notable around the vertebra, the image noise with a grid applied seemed to be somewhat greater at 11% dose reduction at the periphery (Figure 5c) compared with the case without a grid.

## 4 DISCUSSION

In this study, the benefits of a grid applied in DR were evaluated using an adult and a pediatric phantom. For cases with a grid applied, several dose levels were tested: 0% reduction (the same incident dose as the case without a grid) and several levels of dose reduction as determined based on the SIF values that had been preliminarily measured using PMMA phantoms. The adult and pediatric phantom images were visually graded by four radiologists. The results indicate that the quality of images with a grid applied at the 36% dose reduction was slightly superior or practically equal to that without a grid for the adult phantom, and that there is almost no difference in image quality between images with and without a grid at the same incident dose for the pediatric phantom.

For the adult phantom, the visual grading scores were all 3.0 at the same incident dose, whereas a few cases of the score 2.0 were observed at the 36% dose reduction. This result suggests that the use of a grid not only improves the image quality without increasing radiation dose but also hints at the feasibility of reducing the dose, leaving room for some image quality improvement. This can be explained by the notable SNR improvement achieved by the use of a grid when the subject is relatively thick, as reported in some previous studies.^10^, ^11^, ^12^, ^13^, ^14^ For the pediatric phantom, the scores were mostly 2.0, with only one case of 3.0 observed. This suggests that, when a grid is applied, there is no need to increase the radiation dose in order to maintain the receptor dose in consideration of the BF. The result with the pediatric phantom may lead to the question whether the use of a grid is necessary at all because the image quality is not improved at the same incident dose. It should be noted, however, that although the trimmed images used in the visual comparison did not show any notable difference, the contrast of the entire image without a grid was notably degraded by high scatter fraction; the images with a grid applied tended to show correct intensities (contrasts) formed by primary radiation. Therefore, the use of a grid at the same incident dose has the distinct advantage that the images accurately represent the subject's thicknesses and attenuations.

In the present study the approximate dose reduction rates (i.e., 58% and 16% for the adult and the pediatric abdomen, respectively) were determined based on the SIFs measured using the PMMA phantoms with thicknesses equal to the maximum thicknesses of the adult and pediatric phantoms. One of the reasons why these dose reduction rates were not achieved may be the dependency of the detective quantum efficiency on exposure. A decrease in the detector dose because of the use of a grid tends to lower the efficiency of the X‐ray detector.^19^ In addition, it is important to take into consideration the relationship between the PMMA and the human body thicknesses. Since the human body has an elliptical cross‐section, the thickness decreases from the center line of the body to the periphery. Thus, the SIF in the human body depends on the position: it gets lower on the periphery.^14^ Consequently, when the mAs value is reduced according to the dose reduction rate with the maximum thickness, the quality of images with a grid applied at positions away from the center line tends to be lower than that without a grid. In fact, when the images with a grid applied at the reduced dose corresponding to the maximum thickness are compared against those without a grid (Figures 4a vs. d and 5a vs. c), the image noise on the lumber vertebra (located near the center line) was almost the same, whereas in the other positions, it was greater with a grid applied than without a grid. This fact suggests a need to introduce an effective SIF considering the scatter‐to‐primary ratio and also the scatter fraction that varies depending on the position in the human body in future studies.

There are some limitations of this study. First, visual image quality was evaluated using only one type each of the adult and pediatric phantoms. In addition, in the visual assessment, the images were trimmed and the window condition was not varied. These limitations could have affected the visual perception of image quality. Also, further clinical studies would be needed to examine whether the visibility of anatomical structures and the lesion detectability are improved under the same radiation or maintained with reduced radiation doses when using a grid. Second, the assessment of the low contrast detectability was not addressed in this study. The performance can be evaluated with the CDRAD phantom (Artinis Medical Systems, Elst, the Netherlands); however, the analysis is not applicable to evaluate the image with a small FOV corresponding to the pediatric abdomen because the phantom has a size larger than such small FOVs. Furthermore, when a thickness similar to a pediatric abdomen is made with the CDRAD phantom, PMMA slabs are needed; as a result, the phantom shape, like the anthropomorphic phantoms, is not obtained. This is not suitable to the aim of this study. Third, evaluations using chest images that commonly include the wide attenuation differences between lungs and mediastinum were not performed in the present study. A couple of studies reported that the use of a grid decreased SNR only when the area of interest is the lung. One of them reported SIFs of 0.91–0.96 (i.e., dose reduction rates of −9% to −21%) at a position corresponding to the lung region in a phantom made of different simple PMMA parts and aluminum.^13^ However, if the radiation dose is increased according to the SIFs (dose reduction rates) for the lung region, the image quality at the mediastinum might be significantly improved. Thus, an assessment with a more realistic phantom is required for investigating the potential effect of dose reduction when using a grid in chest radiography. Forth, one of the disadvantages of the use of a grid is the emergence of grid‐line artifacts (aliasing of grid lines and a moiré pattern caused by the aliased signals); thus, it is mandatory to introduce a process for suppressing or removing them. While such a process might effectively reduce the grid‐line artifacts, its side effects such as blurring were not investigated in the present study.^16^ The FPD system we used removes the grid pattern using a 2D method,^15^ whereby the filtered area in the frequency domain is limited and the effect of the 2D method on the noise is presumed to be small.

## 5 CONCLUSION

This study demonstrates a potential for improvement in image quality with a grid applied. The result suggests it is unnecessary to increase radiation dose in DR, for both adult (mostly with portable radiography) and pediatric abdomens, when using an anti‐scatter grid. This suggestion needs to be carefully addressed by a study using clinical images.

## AUTHOR CONTRIBUTIONS

Hiroki Kawashima: conceptualization, data curation, investigation, methodology, and writing‐original draft. Katsuhiro Ichikawa: conceptualization, methodology, and writing–review and editing. Azusa Kitao: investigation. Takashi Matsubara: investigation. Takumi Sugiura: investigation. Tomohiro Kobayashi: investigation. Satoshi Kobayashi: Supervision. All authors read and approved the final manuscript.

## CONFLICT OF INTEREST STATEMENT

The authors have no relevant conflicts of interest to disclose.
